# Predictive performances of lipid accumulation product vs. adiposity measures for cardiovascular diseases and all-cause mortality, 8.6-year follow-up: Tehran lipid and glucose study

**DOI:** 10.1186/1476-511X-9-100

**Published:** 2010-09-16

**Authors:** Mohammadreza Bozorgmanesh, Farzad Hadaegh, Fereidoun Azizi

**Affiliations:** 1 Prevention of Metabolic Disorders Research Center, Research Institute for Endocrine Sciences (RIES), Shahid Beheshti University (M.C.), Tehran, Iran; 2 Endocrine Research Center, Research Institute for Endocrine Sciences (RIES), Shahid Beheshti University (M.C.), Tehran, Iran

## Abstract

**Background:**

The body mass index (BMI) is the most commonly used marker for evaluating obesity related risks, however, central obesity measures have been proposed to be more informative. Lipid accumulation product (LAP) is an alternative continuous index of lipid accumulation. We sought in this study to assess if LAP can outperform BMI, waist-to-height-ratio (WHtR), or waist-to-hip-ratio (WHpR) in predicting incident cardiovascular disease (CVD) or all-cause mortality.

**Results:**

Among participants of Tehran Lipid and Glucose Study, 6,751 participants (2,964 men), aged ≥ 30 years, were followed for a median of 8.6 years. We observed 274 deaths (men: 168) and 447 CVD events (men: 257). Levels of common CVD risk factors significantly increased across LAP quartiles. Mortality rates did not differ by LAP quartiles. Among participants free of CVD at baseline [6331 (2,741 men)], CVD incident rates per 1000 person increased in a stepwise fashion with increasing LAP quartile values in both men (from 6.9 to 17.0) and women (from 1.3 to 13.0), (Ps < 0.001).

Among women, a 1-SD increment in log-LAP conferred a 41% increased risk for CVD (HR 1.41, 95% CIs 1.02-1.96). Among men, however, LAP was not observed to be independently associated with increased risk of CVD; except in a sub-group of men assigned to the lifestyle modification interventions, where, LAP predicted CVD risk.

After adjustment with CVD risk factors LAP turned to be inversely associated with risk of all-cause mortality (HR, men 0.74, 95% CIs 0.61-0.90; women, 0.94 95% CIs 0.74-1.20).

Among women, magnitude of increased risk of CVD due to LAP was not different from those of anthropometric measures. Among men, however, WHpR was observed to be more strongly associated with increased risk of CVD than was LAP.

Among neither men nor women were the predictive performances (discrimination, calibration, goodness-of-fit) of the LAP better than those of different anthropometric measures were.

**Conclusions:**

If LAP is to be used for predicting CVD, it might not be superior to WHtR or WHpR.

## Background

In his seminal 1988 Banting award lecture, Reaven introduced insulin resistance as a fundamental "disorder" associated with a set of metabolic abnormalities contributed to the development of cardiovascular disease (CVD) [1]. Large prospective studies [2-7] have shown that insulin resistance is a predictor of coronary artery disease (CAD). It is also relevant to mention that as Reaven found insulin resistant individuals who were not obese, he did not include obesity as a feature of the insulin resistance syndrome. Since then, a plethora of studies increasingly recognized insulin resistance, assessed by various methods, to be underlying factor associated with clustering atherogenic abnormalities [8]. Measuring indices of insulin sensitivity could not be justified, practically in clinical setting. Some organizations, thus, have proposed the "metabolic syndrome (MetS)," a constellation of simple clinical parameters with cut-off values, to find individuals who would probably be insulin resistant [9-14]. Since then, a clinical diagnosis of the MetS was frequently shown to be associated with an increased risk of CVD [8]. It is well-documented that obesity is associated with insulin resistance [8]. However, obesity is remarkably heterogeneous as some obese patients are insulin sensitive whereas others are insulin resistant [15]. With remarkable heterogeneity of obesity in mind, measuring an index of abdominal adiposity such as the waist circumference (WC) is clinically relevant, since among obese individuals, there is a subgroup of abdominally obese patients who are more likely to be insulin resistant [8]. With the introduction of the MetS, the abdominal obesity was recognized as a clinically measurable (although imperfect) entity [8,16-18]. "An Increased WC, however, does not always mean high-risk visceral obesity [8]." WC cannot distinguish visceral adiposity, an important correlate of metabolic abnormalities, from the amount of subcutaneous abdominal fat. On the other hand, as an alternative to MetS, a more fundamental syndromic concept has been introduced. It might be defined by the limited capacity of the human body to buffer and dispose of lipid fuels. During periods of lipid excess, along with expansion of visceral adipocytes, the blood concentrations of certain lipids would become chronically elevated. This state, referred to as "lipid over-accumulation [19] could lead to ectopic deposition of lipids in non-adipose tissues, where insulin resistance and other metabolic dysfunctions would arise [20-22]. Some clinicians therefore used triglyceride (TGs) along with WC (hypertriglyceridemic waist) to find obese patients with abdominal obesity [23]. Lipid accumulation product (LAP), based on a combination of WC and the fasting concentration of circulating TG has recently been introduced by Kahn et al [20]. WC and TGs are each continuously associated with insulin resistance and cardiovascular risk [8]. Against hypertriglyceridemic waist that serve as a dichotomous risk marker [22,23], the LAP was developed to express a continuous risk function [21].

LAP has been shown to predict incident diabetes [24] and all cause mortality [25]. Whether increased LAP confers an excess risk of CVD or not has not directly been addressed. Less is known concerning the performance of LAP as compared to the measures of general and abdominal adiposity. Our primary focus in this study, therefore, was to assess if LAP can outperform BMI, waist-to-height ratio (WHtR), or waist-to-hip ratio (WHpR) in predicting incident CVD.

## Methods

### Study population

Detailed descriptions of the Tehran lipid and glucose study (TLGS) have been reported elsewhere [26]; in brief, the TLGS is a large scale, long term, community-based prospective study performed on a representative sample of residents of district No. 13 of Tehran, capital of Iran. Age and sex distributions of the population in the district were representative of the overall population of Tehran at the time of the baseline examination. A total of 27,340 residents were invited by telephone call, of which 15,005 residents (54.9%) aged ≥3 years participated. The TLGS has two major components: a cross-sectional prevalence study of non-communicable disease and associated risk factors, implemented between March 1999 and December 2001, and a prospective follow-up study. Data collection is ongoing, designed to continue for at least 20 years, at 3-year intervals. Participants were categorized into the cohort (n = 9375) and intervention groups (n = 5630), the latter to be educated for implementation of life style modifications. For the current study, of those aged ≥30 (n = 8,071), we selected those who participated in the follow-up study until 10 March 2009 (n = 7,133). After exclusions (382 missing data), 6,751 (2,964 men) participants remained eligible (response rate 95%). At the time of this study, the median follow up time was 8.7 years. Participants were provided with information regarding the result of their examinations and were given suitable advice. Informed written consent was obtained from all participants and the ethical committee of the Research Institute for Endocrine Sciences approved this study.

### Clinical and laboratory measurements

A trained interviewer collected information using a pretested questionnaire. The information obtained included demographic data, family history of premature CVD, past medical history of CVD, and smoking status. Weight was measured, with subjects minimally clothed without shoes, using digital scales (Seca 707: range 0.1-150 kg) and recorded to the nearest 100 g. Height was measured in a standing position without shoes, using tape meter while shoulders were in a normal alignment. Waist circumference (WC) was measured at the umbilical level and that of the hip at the maximum level over light clothing, using an unstretched tape meter, without any pressure to body surface and measurements were recorded to the nearest 0.1 cm [27]. BMI (kg.m^-2^) was calculated as weight (kg) divided by square of the height (m^2^). WHpR was calculated as WC (cm) divided by hip circumference (cm) and WHtR was calculated as WC divided by height (cm). After a 15-minute rest in the sitting position, two measurements of blood pressure were taken, on the right arm, using a standardized mercury sphygmomanometer (calibrated by the Iranian Institute of Standards and Industrial Researches); the mean of the two measurements was considered as the participant's blood pressure.

A blood sample was drawn between 7:00 and 9:00 AM from all study participants, after 12 to 14 hours overnight fasting. All the blood analyses were undertaken at the TLGS research laboratory on the day of blood collection. Plasma glucose was measured using an enzymatic colorimetric method with glucose oxidase. Fasting plasma glucose (FPG) measurement was performed for all participants, and the standard 2-hour post-challenge plasma glucose (2h-PCPG) test for those not on glucose-lowering drugs. Total cholesterol (TC) was assayed, using the enzymatic colorimetric method with cholesterol esterase and cholesterol oxidase. High-density lipoprotein cholesterol (HDL-C) was measured after precipitation of the apolipoprotein B containing lipoproteins with phosphotungistic acid. TGs were assayed using enzymatic colorimetric assay with glycerol phosphate oxidase. Analyses were performed using Pars Azmon kits (Pars Azmon Inc., Tehran, Iran) and a Selectra 2 auto-analyzer (Vital Scientific, Spankeren, Netherlands). All samples were analyzed when internal quality control met the acceptable criteria. The intra and inter-assay coefficients of variation were both <2.2% for plasma glucose, and 0.5 and 2% for TC, respectively [26].

### Outcome measurements

Details of cardiovascular outcomes have been published elsewhere [28]. In this ongoing study every TLGS' participant is followed up for any medical event during the previous year, by telephone. They are questioned by a trained nurse regarding any medical conditions or whether a related event have occurred, a trained physician collects complementary data during a home visit and/or a visit to the respective hospital to collect data from the participants medical files. In the case of mortality, data are collected from the hospital or the death certificate by an authorized local physician. Collected data are evaluated by an outcome committee consisting of a principal investigator, an internist, an endocrinologist, a cardiologist, an epidemiologist, and the physician who collects the outcome data. Other experts are invited for evaluation of non-communicable disorders, as needed. A specific outcome for each event is assigned according to International Statistical Classification of Diseases and Related Health Problems criteria, 10th Revision, and American Heart Association classification for cardiovascular events [26,29,30]. Coronary heart disease (CHD) includes cases of definite myocardial infarction (MI) diagnosed by electrocardiogram (ECG) and biomarkers, probable MI (positive ECG findings plus cardiac symptoms or signs and biomarkers showing negative or equivocal results), unstable angina pectoris (new cardiac symptoms or changing symptom patterns and positive ECG findings with normal biomarkers), angiographic proven CHD and CHD death. CVD is specified as a composite measure of any CHD events, stroke, or cerebrovascular death.

### Definition of terms

Lipid accumulation product (LAP) is an alternative continuous index of lipid accumulation, which is computed from WC (cm) and TGs (mmol.l^-1^): (WC-65) ×TG (men) and (WC-58) ×TG (women). A previous history of CVD reflected any prior diagnosis of CVD by a physician. The family history of CVD was obtained by asking participants whether any member in their immediate family (first-degree relatives) had experienced a fatal or nonfatal MI, stroke or sudden cardiac arrest. The event was considered premature if it occurred before the age of 55 years in male relatives and before 65 in female relatives [31]. Current smoker was defined as a person who smokes cigarettes daily or occasionally. The diagnosis of hypertension was made in participants who self reported antihypertensive drug usage or in those with the average of the two diastolic blood pressure measurements was ≥ 90 mmHg or when the average of the two systolic blood pressure measurements was ≥ 140 mm Hg [32]. High cholesterol was ascertained in those with total cholesterol ≥ 5.2 mmol.l^-1^, high TGs in those with TGs ≥ 1.7 mmol.l^-1^, and low HDL-C in men with HDL-C < 1.03 mmol.l^-1^l and in women with HDL-C <1.29 mmol.l^-1 ^[33]. Participants using oral hypoglycemic agents or insulin were considered as having diabetes. Diabetes was also ascertained in participants with FPG ≥7.0 mmol.l^-1 ^or 2h-PCPG ≥11.1 mmol.l^-1 ^[34]. Non-HDL- C was calculated by subtracting HDL-C from total cholesterol.

### Statistics analysis

Findings on covariate variables are expressed as means (SD) or percentages for continuously distributed and categorical variables, respectively. We tested for trends across LAP quartiles by using the median in each quartile as a predictor, separately for each sex. The General Linear Model was developed for continuous variables, and the Cox proportional hazards regression model was used for incidence rates. Models were adjusted for age.

For each participant, free of CVD at baseline, the baseline 10-year risk of CVD was calculated using the Framingham's "general CVD risk prediction algorithm [35]." We divided the study sample into those with high and low global risk of CVD. Then, in each sub-group, Kaplan-Meier survival curves were plotted to demonstrate the risk of CVD for each sex across LAP quartiles. The Log-Rank test was performed to examine the significance of trends across LAP quartiles.

When CVD was considered as outcome another 521 prevalent CVD cases were also excluded from the analysis leaving a final sample of 6,331 participants (2741 men). In the analysis of CVD outcome, LAP, BMI, WHpR, and WHtR were assessed using Cox proportional hazards regression analyses. Survival time was the time from start of the follow-up period to the date of the first incident, CVD event, or death due to any cause (failure). The censoring time of an individual was the time from entry into the study to loss to follow-up or the end of the study, whichever happened first. Censored observation meant the subject either refused to participate further in the study (lost to follow-up), died, when death was not the study outcome (competing risk) or continued until the study was ended (administrative censoring). Valid comparison of hazards ratios (HRs) for different continuous measures requires that the units of both variables to be comparable. We, thus, estimated sex-specific hazard ratios (HRs), with 95% confidence intervals (CI) for follow-up events for an one SD increment in natural log-transformed LAP and each respective anthropometric parameter. HRs were adjusted for age, smoking, systolic blood pressure, family history of premature CVD, diabetes, antihypertensive drug usage, HDL and non-HDL cholesterol, FPG, and 2h-PCPG [31], plus the TLGS intervention measures.

We also examined if the effects of LAP on incident CVD or all-cause mortality has been modified by different levels of risk (<20 vs. ≥20%) [35] or life style modification measures. We introduced interaction terms between LAP and global CVD risk levels and LAP and intervention assignment status.

Wald tests of the linear hypotheses concerning the Cox regression models coefficients (paired homogeneity test) were performed to test the null hypotheses that the hazard ratios (effect size) for LAP were equal to those for anthropometric measures.

We compared predictive performance of the LAP with those of the studied anthropometric variables with respect to discrimination, calibration, and goodness-of-fit.

Discrimination is the ability of a prediction model to separate those who develop diabetes events from those who do not and is quantified by the *C *statistic [36]. In the survival analysis, *C *statistic [37] measures the probability that a randomly selected person who developed an event, at the certain specific time has a higher risk score than a randomly selected person who did not develop an event during the same, specific follow-up interval [38].

Calibration, as it is phrased in reference [39] describes how closely predicted probabilities agree numerically with actual outcomes [40,41]. A test very similar to the Hosmer-Lemeshow test has been proposed by Grønnsby and Borgan, which is based on sum of martingale residuals within groups that have been developed by partitioning the data based on the estimated risk score (x'β) [42,43]. Following May and Hosmer we calculated the test statistic using score test (*χ*^*2*^) for the inclusion of G-1 reference cell design variables [41,44,45].

How effectively a model describes the outcome variable is referred to as its goodness-of-fit. Akaike information criterion (AIC) was used as a measure of model fit and informativeness indicating whether the addition of new factors to a base model provides better risk prediction than the base model alone, provided that all of the same individuals are being assessed by both models [46]. When estimating model parameters using maximum likelihood estimation, it is possible to increase the likelihood by adding parameters, which may result in over-fitting. The Bayesian information criterion (BIC) resolves this problem by introducing a penalty term for the number of parameters in the model. It is very closely related to the AIC. In BIC, the penalty for additional parameters is stronger than that of the AIC [47].

Because BMI, WHpR, WHtR, and LAP were highly correlated, we assessed collinearity between these variables using condition indices and variance inflation factor (VIF). Condition indices >30 or VIFs >10 warrant caution [48].

We certify that all applicable institutional and governmental regulations concerning the ethical use of human volunteers were followed during this research. Informed written consent was obtained from all participants and the Ethical Committee of Research Institute for Endocrine Sciences approved this study.

We set the statistical significance level at a two-tailed type I error of 0.05. All statistical analyses were performed using STATA 10.0.

## Result

During follow up (men: 24,209 person-year; women: 31,454 person-year), 274 deaths (men: 168; women: 106) and 447 CVD events (men: 257; women: 190) occurred. The major causes of death were fatal CVD events (36.6%), cancers (11.7%), diabetes complications (4.7%), trauma (4.4%), and infectious disease (3.3). CVD incident rates among participants who were assigned to life style modification measures (men: 10.6, 95% CIs 8.7-13.1; women: 6.2, 95% CIs 4.9-7.8) was not different from those who were not (men: 12.4, 95% CIs 10.6-14.4; women: 6.6, 95% CIs 5.5-7.9). Mortality rates were also the same in the intervention group (men: 6.3, 95% CIs 4.8-8.1; women: 3.6, 95% CIs 2.6-4.8) and the cohort (men: 7.4, 95% CIs 6.1-8.9; women, 3.3, CIs 2.5-4.2) (all Ps > 0.3). The prevalence of high TG, high total cholesterol, low HDL-C, hypertension, diabetes, family history of CVD, and prevalent CVD were 56.6, 58.3, 35.3, 39.0, 16.4, 8.0, and 7.5%, among men and 52.7, 66.1, 25.5, 39.6, 17.7, 12.1, and 5.2% among women, respectively.

As shown in Table 1 and 2, levels of common CVD risk factors significantly increases across LAP quartiles, except for family history of premature CVD among men and smoking among both sexes. CVD incident rates per 1000 person increased in a stepwise fashion with increasing LAP quartile values in both men (from 6.9 to 17.0) and women (from 1.3 to 13.0), (P for trends <0.001). Mortality rates, however, remained unchanged across LAP quartiles.

**Table 1:** Baseline characteristics across quartiles of lipid accumulation product among men.

	**Q1 (cm.mmol.l**^**-1**^**) <2288**	**Q2 (cm.mmol.l**^**-1**^**) 2289-4186**	**Q3 (cm.mmol.l**^**-1**^**) 4192-6900**	**Q4 (cm.mmol.l**^**-1**^**) 6912-57000**	***P *for trends**^**†**^
Number of participants*	741	741	742	740	
Age (years)	48.1(14.4)	49.1(13.6)	50.7(13.0)	49.2(12.5)	
SBP (mmHg)	117.2(19.8)	122.0(19.3)	126.8(20.9)	127.6(19.0)	<0.001
DBP (mmHg)	73.9(11.5)	77.9(10.6)	81.1(11.2)	82.0(11.2)	<0.001
MAP (mmHg)	88.3(12.8)	92.6(12.2)	96.4(13.1)	97.2(12.6)	<0.001
Pulse pressure (mmHg)	43.3(16.1)	44.1(15.0)	45.7(16.0)	45.5(14.1)	<0.001
Pulse (beat per minute)	73.1(10.0)	73.6(9.9)	75.1(9.5)	76.3(9.7)	<0.001
BMI (kg.m^-2^)	22.4(2.8)	25.7(2.7)	27.6(2.8)	29.3(3.5)	<0.001
Waist-to-hip ratio (%)	86.2(5.5)	92.9(5.2)	95.9(5.3)	98.3(5.6)	<0.001
Waist-to-height ratio (%)	46.5(4.6)	52.9(4.4)	56.0(4.4)	58.8(5.2)	<0.001
Lipid accumulation product	11.4(2.2)	35.9(1.2)	60.3(1.2)	117.9(1.4)	<0.001
Triglycerides (mmol.l^-1^)	1.1(1.4)	1.6(1.5)	2.1(1.3)	3.5(1.5)	<0.001
Total cholesterol (mmol.l^-1^)	4.9(1.0)	5.3(0.9)	5.6(1.0)	6.0(1.2)	<0.001
Non-HDL-C (mmol.l^-1^)	3.8(0.9)	4.3(0.9)	4.6(0.9)	5.2(1.1)	<0.001
HDL-C (mmol.l^-1^)	1.1(0.3)	1.0(0.2)	1.0(0.2)	0.9(0.2)	<0.001
FPG (mmol.l^-1^)	5.2(1.4)	5.4(1.5)	5.6(1.7)	6.1(2.3)	<0.001
2h-PCPG (mmol.l^-1^)	5.5(2.6)	6.4(3.2)	6.9(3.5)	8.0(4.2)	<0.001
Assignment to intervention (%)	288 (38.9)	281 (37.9)	275 (37.1)	305 (41.2)	0.347
Diabetes	59 (8.0)	76 (10.3)	146 (19.7)	205 (27.7)	<0.001
Hypertension	253 (34.1)	366 (49.4)	462 (62.3)	476 (64.3)	<0.001
Smoking	253 (34.1)	202 (27.3)	176 (23.7)	208 (28.1)	0.001
Family history of premature CVD	49 (6.6)	58 (7.8)	70 (4.9)	61 (8.2)	0.223
Incident** CHD (95% CIs)	5.3(3.8-7.6)	8.6(6.5-11.5)	11.2(8.7-14.4)	15.0(12.1-18.6)	<0.001
Incident** CVD (95% CIs)	6.9(5.1-9.4)	10.1(7.8-13.2)	13.2(10.4-16.6)	17.0(13.8-20.8)	<0.001
Mortality** rate (95% CIs)	8.3(6.3-11.0)	7.6(5.7-10.2)	6.1(4.4-8.4)	5.8(4.2-8.1)	0.134

BMI, body mass index; DBP, diastolic blood pressure; FPG, fasting plasma glucose; HDL, high density lipoprotein cholesterol; MAP, mean arterial pressure 2h-PCPG, 2-hour post-challenge plasma glucose; SBP, systolic blood pressure;.

Continuous variables are represented as mean (standard deviation) except for triglycerides and lipid accumulation products that are represented as geometric mean (standard deviation of geometric mean). Categorical variables are represented as frequency (percent). Confidence intervals have been provided for incident rates.

**Per 1000 person per year

† P-values were calculated using general linear models, for continuous variables and Cox proportional hazards regression models for incidence rates. Models were sex-specific and age-adjusted.

**Table 2:** Baseline characteristics across quartilesof lipid accumulation product among women.

	**Q1 (cm.mmol.l**^**-1**^**) <2752**	**Q2 (cm.mmol.l**^**-1**^**) 2755-5040**	**Q3 (cm.mmol.l**^**-1**^**) 5043-8265**	**Q4 (cm.mmol.l**^**-1**^**) 8268-54948**	***P *for trends**^**†**^
Number of participants*	947	954	939	947	
Age (years)	40.6(10.4)	46.5(11.7)	49.9(11.6)	52.1(10.8)	
SBP (mmHg)	112.7(15.7)	120.9(18.7)	127.7(21.8)	133.2(21.9)	<0.001
DBP (mmHg)	74.6(9.4)	78.3(10.1)	82.0(10.7)	84.3(10.8)	<0.001
MAP (mmHg)	87.3(10.5)	92.5(11.8)	97.2(13.1)	100.6(13.2)	<0.001
Pulse pressure (mmHg)	38.1(11.8)	42.5(14.5)	45.7(16.8)	48.9(16.8)	<0.001
Pulse (beat per minute)	80.9(11.9)	81.0(12.1)	80.8(11.9)	81.7(12.0)	<0.012
BMI (kg.m^-2^)	24.7(3.5)	27.9(3.6)	29.9(4.2)	31.7(4.5)	<0.001
Waist-to-hip ratio (%)	78.5(6.5)	85.3(6.6)	88.5(6.8)	92.2(7.0)	<0.001
Waist-to-height ratio (%)	49.9(5.2)	57.1(5.4)	61.3(5.9)	65.4(6.2)	<0.001
Lipid accumulation product	17.8(1.6)	42.9(1.2	73.0(1.2)	135.6(1.4)	<0.001
Triglycerides (mmol.l^-1^)	1.0(1.4)	1.4(1.3)	2.0(1.3)	3.2(1.4)	<0.001
Total cholesterol (mmol.l^-1^)	5.0(1.0)	5.5(1.0)	6.0(1.1)	6.5(1.3)	<0.001
Non-HDL-C (mmol.l^-1^)	3.8(0.9)	4.3(1.0)	4.9(1.1)	5.5(1.3)	<0.001
HDL-C (mmol.l^-1^)	1.3(0.3)	1.2(0.3)	1.1(0.3)	1.0(0.3)	<0.001
FPG (mmol.l^-1^)	5.0(1.3)	5.3(1.6)	5.9(2.3)	6.5(2.8)	<0.001
2h-PCPG (mmol.l^-1^)	5.8(1.8)	6.5(2.3)	7.4(3.2)	8.7(4.2)	<0.001
Assignment to intervention (%)	362 (38.2)	387 (40.6)	363 (38.7)	365 (38.5)	0.657
Diabetes	46 (4.9)	104 (10.9)	197 (21.0)	322 (34.0)	<0.001
Hypertension	288 (28.3)	472 (42.5)	585 (62.3)	699 (73.8)	<0.001
Smoking	44 (4.7)	37 (3.9)	30 (3.2)	28 (3.0)	0.748
Family history of premature CVD	92 (9.7)	107 (11.2)	129 (13.7)	131 (13.8)	<0.010
Incident** CHD (95% CIs)	1.1 (0.6-2.2)	3.6 (2.4-5.2)	7.4 (5.7-9.7)	11.2 (8.9-14.0)	<0.001
Incident** CVD (95% CIs)	1.3 (0.7-2.4)	4.1 (2.9-5.8)	8.3 (6.4-10.7)	13.0 (10.5-16.0)	<0.001
Mortality** rate (95% CIs)	2.4(1.5-3.7)	2.5(1.6-3.9)	4.1(2.9-5.8)	4.5(3.3-6.3)	0.673

BMI, body mass index; DBP, diastolic blood pressure; FPG, fasting plasma glucose; HDL, high density lipoprotein cholesterol; MAP, mean arterial pressure 2h-PCPG, 2-hour post-challenge plasma glucose; SBP, systolic blood pressure;.

Continuous variables are represented as mean (standard deviation) except for triglycerides and lipid accumulation products that are represented as geometric mean (standard deviation of geometric mean). Categorical variables are represented as frequency (percent). Confidence intervals have been provided for incident rates.

**Per 1000 person per year

† P-values were calculated using general linear models, for continuous variables and Cox proportional hazards regression models for incidence rates. Models were sex-specific and age-adjusted.

Table 3 presents the contribution of LAP to the risk of CVD, independent of other CVD risk factors, separately for men and women.

**Table 3:** Contribution of LAP to the risk of CVD, independent of common CVD risk factors among men and women.

	Men		Women	
	
	HR* (95% CIs)	P-value	HR* (95% CIs)	P-value
Age (years)	1.05 (1.04-1.06)	<0.001	1.06 (1.04-1.08)	<0.001
Smoking	1.86 (1.37-2.51)	<0.001	2.97 (1.36-6.47)	0.006
Premature history of CVD	1.47 (0.94-2.32)	0.094	1.60 (1.01-2.52)	0.044
Diabetes	1.30 (0.81-2.08)	0.271	1.95 (1.13-3.35)	0.017
Antihypertensive drug use	1.42 (0.97-2.08)	0.068	2.04 (1.40-2.98)	<0.001
Systolic blood pressure (mmHg)	1.40 (1.24-1.58)	0.000	1.23 (1.04-1.46)	0.014
Intervention	0.76 (0.57-1.01)	0.059	0.96 (0.67-1.38)	0.842
Non-HDL-C (mmol.l^-1^)	1.34 (1.15-1.56)	<0.001	1.25 (1.05-1.50)	0.014
HDL-C (mmol.l^-1^)	0.81 (0.67-0.97)	0.022	1.06 (0.88-1.28)	0.547
FPG (mmol.l^-1^)	1.29 (0.97-1.71)	0.075	0.81 (0.60-1.09)	0.169
2h-PCPG (mmol.l^-1^)	0.97 (0.79-1.20)	0.800	1.10 (0.90-1.35)	0.342
Ln _LAP _(cm.mmol.l^-1^)	1.06 (0.89-1.26)	0.535	1.41 (1.02-1.96)	0.038

* For one SD increment in each continuous predictor, obtained from multivariate Cox proportional hazards regression model adjusted for age, smoking, systolic blood pressure, family history of premature CVD, diabetes, antihypertensive drug usage, HDL and non-HDL cholesterol, FPG, and 2h-PCPG , plus the TLGS intervention measures.

CVD, cardiovascular disease; FPG, fasting plasma glucose; HDL-C, high density lipoprotein cholesterol; HR, hazard ratio; LAP, lipid accumulation product; Ln, naturally logarithmically transformed; 2h-PCPG, 2-hour post-challenge plasma glucose;

Among women, a 1-SD (0.81) increment in log LAP conferred a 41% increased risk for CVD (HR 1.41, 95% CI 1.02-1.96). Among men, however, LAP was not observed to be independently associated with increased risk of CVD. Multivariate adjusted hazard ratios for a 1-SD increment in WC for CVD and all-cause mortality among men were 1.18 (95% CIs 1.01-1.39, P = 0.036) and 0.90 (95% CIs 0.72-0.1.12, P = 0.348), respectively; the corresponding figures among women were 1.24 (95% CIs 1.03-1.49, P = 0.021) and 0.84 (95% CIs 0.65-1.09, P = 0.197). Hazard ratios for a 1-SD increment in TG for CVD and all-cause mortality among men were 1.00 (95% CIs 0.88-1.1.41, P = 0.953) and 0.75 (95% CIs 0.56-1.00, P = 0.055), respectively; the corresponding figures among women were 0.98 (95% CIs 0.80-1.20, P = 0.834) and 1.26 (95% CIs 0.88-1.81, P = 0.206), respectively.

We failed to demonstrate any increased risk of all-cause mortality owing to LAP (Table 4). In fact, after adjustment with CVD risk factors LAP turned to be inversely associated with risk of all-cause mortality. This association, however, was statistically significant among men (HR 0.74, 95% CIs 0.61-0.90) but not among women (HRs 0.94 95% CIs 0.74-1.20).

**Table 4:** Contribution of LAP to the risk of all-cause mortality, independent of common CVD risk factors among men and women.

	Men		Women	
	
	HR* (95% CIs)	P-value	HR* (95% CIs)	P-value
Age (years)	1.09 (1.07-1.11)	<0.001	1.14 (1.11-1.18)	<0.001
Smoking	2.01 (1.32-3.04)	0.001	2.27 (0.69-7.50)	0.179
Premature history of CVD	0.58 (0.23-1.41)	0.228	1.16 (0.55-2.47)	0.692
Diabetes	1.52 (0.86-2.69)	0.151	1.30 (0.51-3.34)	0.579
Antihypertensive drug use	1.97 (1.31-2.98)	0.001	1.59 (0.92-2.74)	0.094
Systolic blood pressure (mmHg)	1.20 (1.03-1.40)	0.022	1.03 (0.80-1.32)	0.822
Intervention	0.69 (0.47-1.02)	0.061	1.10 (0.66-1.84)	0.708
Non-HDL-C (mmol.l^-1^)	0.94 (0.75-1.18)	0.595	0.86 (0.64-1.15)	0.295
HDL-C (mmol.l^-1^)	0.87 (0.69-1.08)	0.197	0.93 (0.71-1.22)	0.582
FPG (mmol.l^-1^)	1.19 (0.82-1.72)	0.366	0.67 (0.34-1.32)	0.253
2h-PCPG (mmol.l^-1^)	1.02 (0.79-1.31)	0.892	1.18 (0.86-1.63)	0.310
Ln _LAP _(cm.mmol.l^-1^)	0.74 (0.61-0.90)	0.003	0.88 (0.60-1.30)	0.530

* For one SD increment in each continuous predictor, obtained from multivariate Cox proportional hazards regression model adjusted for age, smoking, systolic blood pressure, family history of premature CVD, diabetes, antihypertensive drug usage, HDL and non-HDL cholesterol, FPG, and 2h-PCPG , plus the TLGS intervention measures.

**Figure 1:**
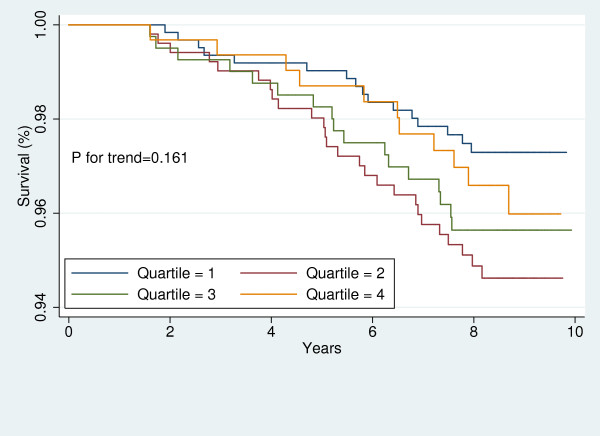
**Kaplan-Meier curves for incident CVD across LAP quartiles in men with global CVD risk less than 20%**.

**Figure 2:**
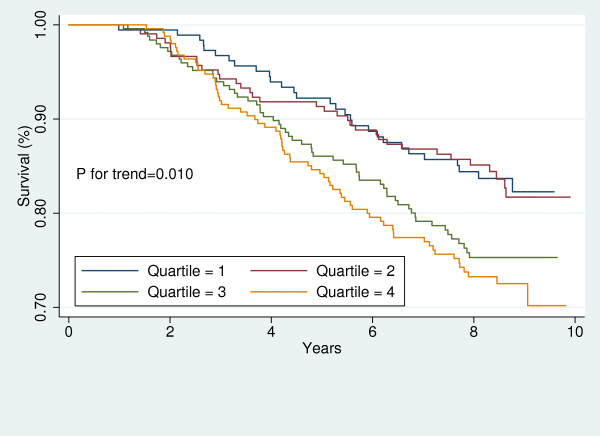
**Kaplan-Meier curves for incident CVD across LAP quartiles in men with global CVD risk 20% or more**.

**Figure 3:**
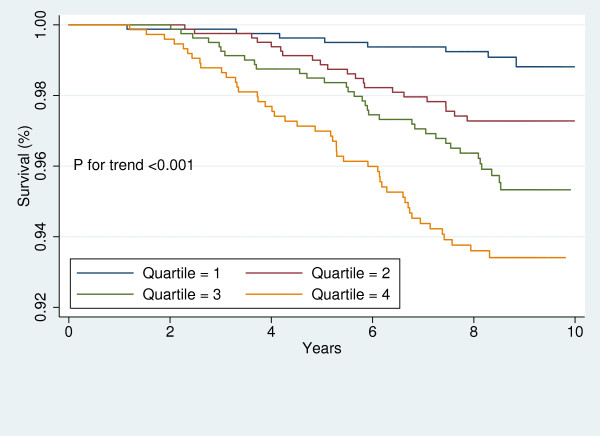
**Kaplan-Meier curves for incident CVD across LAP quartiles in women with global CVD risk less than 20%**.

**Figure 4:**
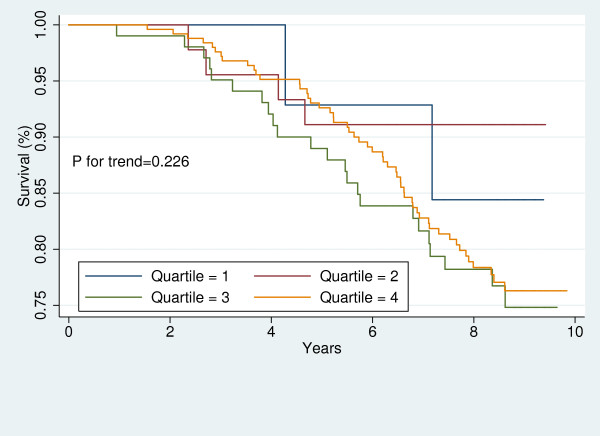
**Kaplan-Meier curves for incident CVD across LAP quartiles in women with global CVD risk 20% or more**.

**Figure 5:**
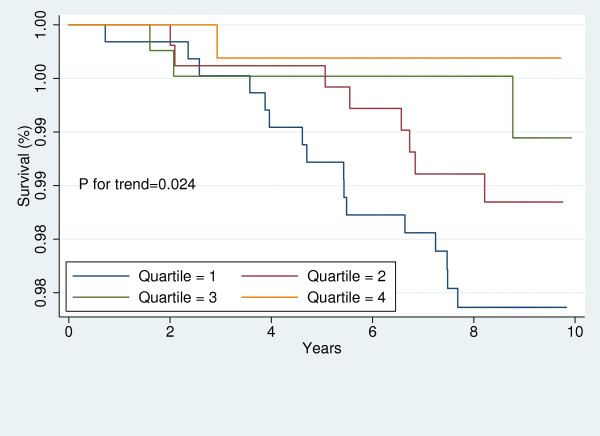
**Kaplan-Meier curves for all-cause mortality across LAP quartiles in men with global CVD risk less than 20%**.

**Figure 6:**
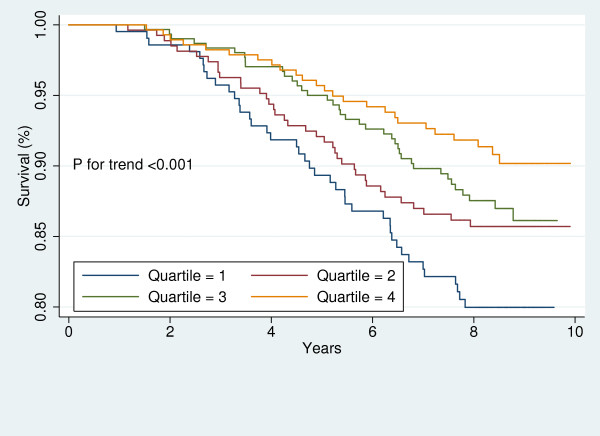
**Kaplan-Meier curves for all-cause mortality across LAP quartiles in men with global CVD risk 20% or more**.

**Figure 7:**
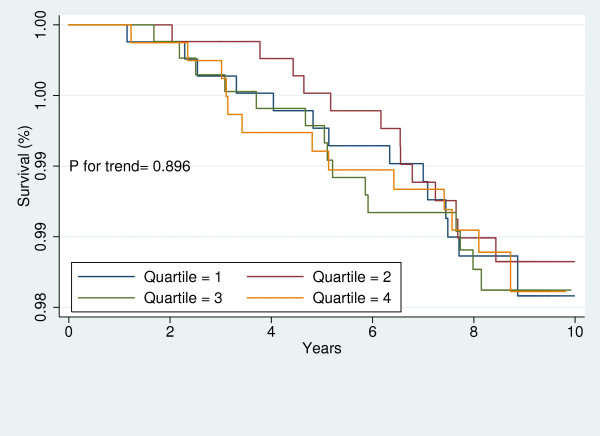
**Kaplan-Meier curves for all-cause mortality across LAP quartiles in women with global CVD risk less than 20%**.

**Figure 8:**
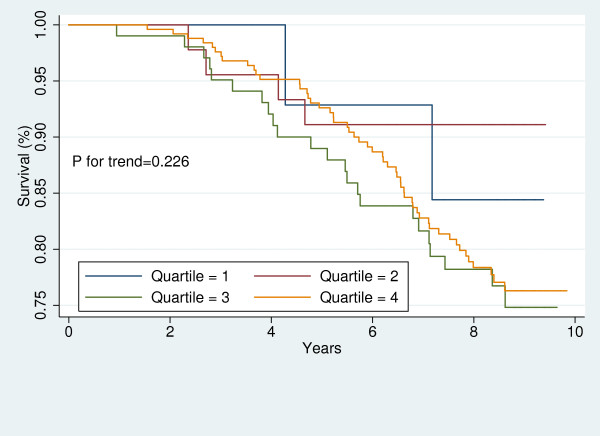
**Kaplan-Meier curves for all-cause mortality across LAP quartiles in women with global CVD risk 20% or more**.

CVD, cardiovascular disease; FPG, fasting plasma glucose; HDL-C, high density lipoprotein cholesterol; HR, hazard ratio; LAP, lipid accumulation product; Ln, naturally logarithmically transformed; 2h-PCPG, 2-hour post-challenge plasma glucose;

Figures 1, 2, 3, 4, 5, 6, 7, and 8 represent the sex-specific Kaplan-Meier curves for CVD and all-cause mortality, across LAP quartiles in the two sub-groups defined by baseline global CVD risk ≥ 20% and < 20%. Among women with global CVD risk less than 20%, the probability of remaining free of CVD decreased in stepwise fashion across LAP quartiles. Such a trend was not observed among women with global CVD risk of 20% or more. In the contrary, in men it was among those with global CVD risk ≥20% that the probability of remaining free of CVD across decreased across LAP quartiles. Kaplan-Meier survival curves plotted for all-cause mortality showed that survival prognosis improved in stepwise fashion across LAP quartiles among men, regardless of their baseline global CVD risk. Among women survival prognosis did not differ by LAP quartiles.

In multivariate-adjusted Cox proportional hazard regression models LAP predicted incident CVD only among women with global CVD risk less than 20%, while it did not predict all-cause mortality in any of sub-groups (Table 5).

**Table 5:** Contribution of LAP to the risk of CVD and all-cause mortality, independent of common CVD risk factors.

		Men		Women	
		HR* (95% CIs)	P-value	HR* (95% CIs)	P-value
CVD	Global CVD risk^† ^<20%	1.07 (0.80-1.42)	0.643	1.46 (1.05-2.03)	0.025
	Global CVD risk^† ^≥20%	1.09 (0.90-1.32)	0.374	1.20 (0.75-1.92)	0.440
All-cause mortality	Global CVD risk^† ^<20%	0.75 (0.52-1.08)	0.128	0.74 (0.48-1.15)	0.183
	Global CVD risk^† ^≥20%	0.80 (0.62-1.01)	0.064	1.17 (0.54-2.57)	0.688
CVD	Assigned to the intervention	1.58 (1.11-2.26)	0.011	1.43 (0.89-2.29)	0.143
	Not assigned to the intervention	1.01 (0.82-0.1.25)	0.896	1.33 (0.90-1.96)	0.148
All-cause mortality	Assigned to the intervention	0.71 (0.49-1.02)	0.061	1.16 (0.62-2.15)	0.641
	Not assigned to the intervention	0.77 (0.61-0.98)	0.034	0.73 (0.44-1.20)	0.214

* For one SD increment in each continuous predictor, obtained from multivariate Cox proportional hazards regression model adjusted for age, smoking, systolic blood pressure, family history of premature CVD, diabetes, antihypertensive drug usage, HDL and non-HDL cholesterol, FPG, and 2h-PCPG , plus the TLGS intervention measures.

†calculated using "the Framingham general CVD prediction algorithm". (D'Agostino RB, Sr., Vasan RS, Pencina MJ, Wolf PA, Cobain M, Massaro JM, Kannel WB: **General Cardiovascular Risk Profile for Use in Primary Care: The Framingham Heart Study**. *Circulation *2008, **117**:743-753)

CVD, cardiovascular disease; FPG, fasting plasma glucose; HDL-C, high density lipoprotein cholesterol; HR, hazard ratio; LAP, lipid accumulation product; Ln, naturally logarithmically transformed; 2h-PCPG, 2-hour post-challenge plasma glucose;

When we introduced interaction terms between intervention assignment status and LAP to the models, among both men and women the results remained essentially unchanged (Ps for interactions >0.07). Nonetheless, we examined the effects of LAP on risk of CVD and all-cause mortality separately for those who were assigned to the life style modification measures and those who were not (Tables 5). The finding of interest was that LAP conferred a 58% increase in the risk of CVD among men who were assigned to the life style modification interventions (HR 1.58, 95% CIs 1.11-2.26). Among women CVD risk conferred by LAP for both sub-groups were similar to that of whole sample, though, no longer statistically significant. It may be due to the broadened 95% CIs and decreased statistical power.

Relative importance of LAP has been compared with those of anthropometric measures in Table 6. Among women, the magnitude of increased risk of CVD due to LAP was not different from those of anthropometric measures. Among men, however, WHpR was observed to be more strongly associated with increased risk of CVD than was LAP.

**Table 6:** Predictive accuracy of the LAP as compared with those of BMI, WHpR, and WHtR.

Factor		HR* (95% CIs)	**Wald Χ**^**2 **^**(P value)**^**†**^	AIC	BIC	**Discrimination**^**¥**^	**Calibration**^**¶**^
Male							
	LAP	1.06 (0.89-1.26)	-	3134	3204	0.785 (0.756-0.813)	21.6 (0.010)
	BMI	1.12 (0.94-1.33)	0.51 (0.474)	3133	3203	0.785 (0.759-0.811)	18.4 (0.031)
	WHpR	1.25 (1.06-1.47)	4.62 (0.032)	3128	3198	0.788 (0.762-0.814)	18.0 (0.035)
	WHtR	1.20 (1.00-1.43)	2.69 (0.101)	3131	3201	0.787 (0.759-0.814)	15.1 (0.088)
Female							
	LAP	1.41 (1.02-1.96)	-	1876	1947	0.846 (0.818-0.875)	12.2 (0.201)
	BMI	1.15 (0.97-1.37)	0.81 (0.367)	1878	1951	0.847 (0.825-0.868)	12.8 (0.170)
	WHpR	1.35 (1.10-1.65)	0.13 (0.718)	1872	1945	0.848 (0.820-0.876)	11.9 (0.218)
	WHtR	1.30 (1.08-1.57)	0.17 (0.680)	1873	1946	0.849 (0.825-0.873)	12.6 (0.180)

* Hazard ratios for one SD increment in each anthropometric variable and one SD increment in log LAP level, obtained from multivariate Cox proportional hazard regression model adjusted for age, smoking, systolic blood pressure, family history of premature CVD, diabetes, smoking, antihypertensive drug usage, HDL and non-HDL cholesterol, FPG, and 2h-PCPG , plus the TLGS intervention measures

† Wald tests Χ^2 ^(P value) of the linear hypotheses concerning the Cox regression models coefficients (paired homogeneity test) were performed to test the null hypotheses that the hazard ratios (effect size) for LAP were equal to those for BMI, WHpR, or WHtR.

¥ Assessed with C statistic (95% CIs)

¶ Assessed with Hosmer-Lemeshow Χ^2 ^(P value)

Abbreviations: AIC: Akaike information criteria; BIC: Bayesian information criteria; BMI: body mass index; LAP: lipid accumulation product; WHpR: waist-to-hip ratio; WHtR: waist-to-height ratio;

Standard deviations: Men → LAP (mmol.cm) (naturally logarithmically-transformed),1.15; BMI (kg.m^-2^), 3.9; WHpR (%)7.0;WHtR 6.5 (%)

Standard deviations: Women→ LAP (mmol.cm) (naturally logarithmically-transformed), 0.81; BMI (kg.m^-2^), 4.7; WHpR (%)8.3;WHtR 8.1 (%)

Table 6 represents the different measures of predictive performance of models incorporating LAP, BMI, WHpR, and WHtR, each at a time. Models incorporating LAP and different anthropometric measures generally better fit the data among women (AICs 1872-1878, BIC 1945-1951) than among men (AICs 3128-3134, BIC 3198-3204). The discrimination capacities of the models were also higher among women (C statistics 0.846-0.849) than among men (C statistics 0.785-0.788). The model-based estimated risk of CVD again better agreed with observed risk among women (Hosmer-Lemeshow χ^2 ^11.9-12.8) than among men (Hosmer-Lemeshow χ^2 ^18.0-21.6). Among neither men nor women were the predictive performances of the LAP better than those of different anthropometric measures were.

## Discussion

For the first time, using data from a community-based prospective study of men and women for a median of 8.6 years, we demonstrated that the LAP was independently associated with an increased risk of incident CVD among women and that the magnitude of this risk was not significantly higher than those conferred by BMI, WHpR, or WHtR. Among men, after controlling common CVD risk factors, we observed that LAP was not associated with any significant increased risk of incident CVD. The only exception observed was among a subgroup of men who were assigned to the life style modification interventions. In this sub-group a 1-SD increment in naturally logarithmatically transformed LAP conveyed a 58% increased CVD risk. Among men the magnitude of risk conferred by WHpR exceeded that of LAP. No associations were observed between the LAP and increased risk of all-cause mortality in men or women.

As a component of a multivariate predictive model for incident CVD, the LAP, BMI, WHpR, and WHtR had the same performance with respect to the discrimination capacity and the calibration. Trade-offs between bias and variance (AIC and BIC), due to incorporating LAP into multivariate predictive models was similar to those due to BMI, WHpR, or WHtR.

Some studies recently compared different anthropometric measures in terms of their ability to predict CVD. They observed that measures of central and overall adiposity predicted CVD to a similar degree except for slight superiority for WHpR [49-52].

Nearly one-third of the population in the industrialized world are currently affected by obesity [53]. Whether obesity exerts independent and direct effects on CVD beyond its strong association with established clinical risk factors remains controversial [54]. Currently used risk functions for the prediction of coronary events in the general population do not include measures of excess body weight because it is considered to affect risk indirectly through more proximal physiological and metabolic factors such as blood pressure, lipid levels, and diabetes [55]. Traditionally, anthropometric measures such as BMI or WC have been used to quantify adiposity. Results from the present study support other studies that have found that "obesity predicts risk of CVD incidence beyond the established clinical conditions" [54]. Abdominal visceral adipose tissue (VAT) has been highlighted as unique, pathogenic fat depots [56,57]. VAT is hypothesized to have a systemic effect on atherosclerosis. LAP, has been reported to offer an inexpensive and non invasive tool to estimate total body lipid accumulation in comparison with sophisticated imaging methods for estimating the lipid burden or uptake in isolated tissues [21]. The fact that, unlike LAP, the MetS components are not used as continuous variables makes these screening tools less than ideal for the optimal diagnosis of the cardio-metabolic risk [8]. It is commonly believed that only new calculators providing a continuous score could address this issue [58] and LAP has been developed on such a background. We observed, however, that if LAP is to be used for predicting CVD, it might not be superior to WHtR or WHpR.

Our results confirm the association of LAP with CVD risk factors. LAP increased risk of incident CVD among women. The same finding has been previously reported [59]. LAP predicted CVD risk only among men who were assigned to the lifestyle modification interventions. LAP may not predict CVD risk among men who are already at increased risk for CVD. We failed to demonstrate any association with increased risk of all-cause mortality due to LAP. In fact, after adjustment with CVD risk factors, LAP came to be inversely associated with risk of all-cause mortality. This association, however, was statistically significant among men (HR 0.74, 95% CIs 0.61-0.90) but not among women (HRs 0.94 95% CIs 0.74-1.20). This negative confounding would not be underappreciated. No clear-cut explanation, though, is readily available and confirmation in an independent study is required before this surprising observation could be understood. There is, to our knowledge, only one other study (PreCIS data base study), in which LAP has been found to be independently associated with increased mortality in both sexes. In the PreCIS population, LAP was reported to be associated with all-cause mortality and the association was stronger among women than among men [59]. The proportion of CVD deaths in all deaths can explain the discrepancy, since only 36.6% of deaths in the TLGS were attributable to the CVD while the PreCIS population is known to be at increased risk of mortality from CVD. As compared to the TLGS, the PreCIS population were older (mean age 55 years) and at a greater risk of CVD; the prevalence of hypertension, diabetes, family history of CVD, and CVD were 65, 19, 42, and 59% respectively [59]. The corresponding figures in the TLGS population were 24, 17.1, 6.2, and 10.3%, respectively. Differences in the baseline LAP from one cohort to another may also account for different findings. Other possible explanations may be the covariance of obesity with other CVD risk factors, unmeasured confounders, or misclassification bias from use of surrogate markers of obesity [53,56].

WC was not associated with increased all-cause mortality among the TLGS' men or women. In previous studies showing the association between WC and mortality, the association was stronger for CVD mortality than overall mortality [59]. Among men, we observed TG to be inversely associated with all-cause mortality (HR 0.75, 95% CIs 0.56-1.00); WC showed no association with mortality (HR 0.90, 95% CIs 0.72-1.12). Difference in methods used to measure WC in different studies could at least partly describe the difference in the results obtained. The consequences of elevated TGs are controversial and the benefit of reducing these levels is yet to be clarified. Inverse or lack of association between TGs levels and all-cause mortality has previously been reported [60,61]. Men have been shown to be resistive to hazards of adiposity; and mild-grade central obesity has been reported to be protective [51]. Lower TGs levels resulting from malnourishment, combined with lower WC due to recent weight loss related to chronic illness might have biased our findings. In developing countries mortalities are still more likely to be due to chronic inflammations, undernourishment, or cancers than CVDs, and therefore, may be less likely to be associated with CVD risk factors including LAP [62,63]. Some investigators argued that introduction of both HDL-C and TGs as independent covariates in a model is inappropriate owing to multicollinearity and an intimate correlation between these variables in lipid metabolism [64]. However, even after we excluded HDL-C from analyses the results remained essentially unchanged (data not shown).

Strength of the present study lies in its prospective nature, the use of a large population-based-cohort of both sexes, accurate and valid data on risk factors at baseline, continuous surveillance of mortality and CVD events based on standard criteria.

Some limitations to our study merit mentioning. First, in this study, no data was available about TGs lowering drugs. Second, the small number of incident events precluded stratification of analyses by age. Third, sub-group samples might not have enough statistical power to detect effects. Finally, the population studied was of Persian ancestry, our results, thus, cannot be readily extrapolated to other populations.

## Conclusion

We demonstrated that the LAP was independently associated with an increased risk of incident CVD among women and that the magnitude of this risk due to LAP was not significantly higher than those due to BMI, WHpR, or WHtR. However, among men, after controlling common CVD risk factors, we observed that LAP was not associated with any significant increased risk of incident CVD, except for those assigned to the lifestyle modification interventions. Among men, the magnitude of risk conferred by WHpR exceeded that conferred by LAP. No association was observed between the LAP and increased risk of all-cause mortality in women. Among men, LAP was inversely associated with all-cause mortality. If LAP is to be used for predicting CVD, it might not be superior to WHtR or WHpR.

## Competing interests

The authors declare that they have no competing interests.

## Authors' contributions

MB designed the study, performed the statistical analysis, interpreted the analyses and drafted the manuscript. FH interpreted the analyses and revised the manuscript critically for important intellectual content. FA revised the manuscript critically for important intellectual content. All authors read and approved the final manuscript.
